# Expression of TMEM106B, the frontotemporal lobar degeneration-associated protein, in normal and diseased human brain

**DOI:** 10.1186/2051-5960-1-36

**Published:** 2013-07-11

**Authors:** Johanna I Busch, Maria Martinez-Lage, Emily Ashbridge, Murray Grossman, Vivianna M Van Deerlin, Fenghua Hu, Virginia MY Lee, John Q Trojanowski, Alice S Chen-Plotkin

**Affiliations:** 1 Departments of Neurology, Perelman School of Medicine at the University of Pennsylvania, Philadelphia, PA, USA; 2 Departments of Pathology and Laboratory Medicine, Perelman School of Medicine at the University of Pennsylvania, Philadelphia, PA, USA; 3 Department of Molecular Biology and Genetics, Cornell University, Ithaca, NY, USA; 4 Department of Neurology, Perelman School of Medicine, 3610 Hamilton Walk, 166 Johnson Pavilion, Philadelphia, PA 19104, USA; 5 Pathology and Laboratory Medicine, The Hospital of the University of Pennsylvania, 3400 Spruce Street, 6 Founders, Philadelphia, PA 19104, USA; 6 Department of Neurology, The Hospital of the University of Pennsylvania, 3400 Spruce Street, 2 Gibson, Philadelphia, PA 19104, USA; 7 Pathology and Laboratory Medicine, Perelman School of Medicine at the University of Pennsylvania, 3400 Spruce Street, 7.103 Founders Pavilion, Philadelphia, PA 19104, USA; 8 Department of Molecular Biology and Genetics, Weill Institute for Cell and Molecular Biology, Cornell University, 345 Weill Hall, Ithaca, USA; 9 University of Pennsylvania, 3600 Spruce Street, 3rd Floor Maloney Building, Philadelphia, PA 19104, USA

**Keywords:** *TMEM106B*, Frontotemporal lobar degeneration, Frontotemporal dementia, TDP-43, Progranulin, FTLD-TDP

## Abstract

**Background:**

Frontotemporal lobar degeneration (FTLD) is the second most common cause of dementia in individuals under 65 years old and manifests as alterations in behavior, personality, or language secondary to degeneration of the frontal and/or temporal lobes. FTLD-TDP, the largest neuropathological subset of FTLD, is characterized by hyperphosphorylated, ubiquitinated TAR DNA-binding protein 43 (TDP-43) inclusions. Mutations in progranulin (*GRN*), a neuroprotective growth factor, are one of the most common Mendelian genetic causes of FTLD-TDP. Moreover, a recent genome-wide association study (GWAS) identified multiple SNPs within the uncharacterized gene *TMEM106B* that significantly associated with FTLD-TDP, suggesting that *TMEM106B* genotype confers risk for FTLD-TDP. Indeed, TMEM106B expression levels, which correlate with *TMEM106B* genotype, may play a role in the pathogenesis of disease.

**Results:**

Since little is known about TMEM106B and its expression in human brain, we performed immunohistochemical studies of TMEM106B in postmortem human brain samples from normal individuals, FTLD-TDP individuals with and without *GRN* mutations, and individuals with other neurodegenerative diseases. We find that TMEM106B protein is cytoplasmically expressed in both histopathologically affected and unaffected areas of the brain by neurons, glia, and endothelial cells/pericytes. Furthermore, we demonstrate that TMEM106B expression may differ among neuronal subtypes. Finally, we show that TMEM106B neuronal expression is significantly more disorganized in FTLD-TDP cases with *GRN* mutations, compared to normal and disease controls, including FTLD-TDP cases without *GRN* mutations.

**Conclusions:**

Our data provide an initial neuropathological characterization of the newly discovered FTLD-TDP-associated protein TMEM106B. In addition, we demonstrate that FTLD-TDP cases with *GRN* mutations exhibit a loss of neuronal TMEM106B subcellular localization, adding to evidence that TMEM106B and progranulin may be pathophysiologically linked in FTLD-TDP.

## Background

Frontotemporal lobar degeneration (FTLD) is a fatal neurodegenerative disease characterized by selective degeneration of the frontal and temporal lobes [1,2]. Functionally, patients often present with alterations in behavior, personality and language, rather than with memory impairment as seen in patients with Alzheimer’s disease [3-5]. FTLD is neuropathologically classified into two major subtypes: FTLD-tau and FTLD-TDP [4,6]. FTLD-tau cases are characterized by abnormal accumulations of the microtubule-associated protein tau in neurons and glia, while FTLD-TDP cases harbor neuronal inclusions of ubiquitinated, hyperphosphorylated TAR DNA-binding protein 43 (TDP-43) [7,8].

FTLD-TDP comprises approximately 50% of clinical FTLD [9]. Mutations in the progranulin gene (*GRN*), which codes for a growth factor with neuroprotective effects [10,11] account for ~10% of FTLD-TDP [12-14]. The majority of these autosomal dominant mutations result in premature termination codons and thus progranulin haploinsufficiency [14,15]. In addition, expansions in the *C9orf72* gene have recently been shown to be an important Mendelian cause of FTLD-TDP [16,17]. However, the majority of FTLD-TDP cases do not show clear Mendelian patterns of inheritance.

In order to identify additional genetic risk factors, we previously performed a genome-wide association study (GWAS) and identified multiple SNPs within the uncharacterized gene *TMEM106B* that significantly associated with FTLD-TDP (odds ratio 1.6, p = 1.08 × 10^-11^ for top SNP rs1990622) [18]. This association has been replicated in a clinically diagnosed cohort of patients [19] and was most recently replicated in a cohort of FTLD-TDP patients carrying *GRN* mutations [20], although other investigators have not replicated the association [21]. We and others have investigated the physiological [22-24] and pathophysiological [25-28] function of TMEM106B. *TMEM106B* genetic variants may confer increased disease risk by increasing levels of TMEM106B expression, since mRNA expression levels of *TMEM106B* are >2.5-fold higher in FTLD-TDP cases vs. controls [18], and are particularly increased in FTLD-TDP cases with *GRN* mutations [24]. Moreover, *TMEM106B* risk genotypes have been associated with higher levels of TMEM106B expression in lymphoblastoid cell lines [29] and in human brain tissue [18,24], suggesting that the variants found by GWAS tag a *cis*-acting mechanism for regulating TMEM106B expression. One possible mechanism was recently identified by Nicholson et al. [20], who demonstrated that differential isoforms at the coding SNP rs3173615 (p.T185S), which is in linkage disequilibrium with the GWAS SNP rs1990622, result in different rates of protein degradation. The risk (T185) isoform of TMEM106B is degraded less quickly than the protective (S185) form of TMEM106B. Together, these data suggest that *TMEM106B* variants resulting in higher levels of TMEM106B protein may increase disease risk.

Evidence further suggests that *TMEM106B* risk genotypes/increased TMEM106B expression may modulate disease risk by affecting progranulin pathways. For example, *TMEM106B* risk genotypes have been associated with decreased plasma progranulin levels [26], and significantly earlier onset of disease in *GRN* mutation carriers [27]. Moreover, TMEM106B, which localizes to late endosomes / lysosomes in multiple cell lines and in mouse primary cortical and hippocampal neurons [22-24], has been shown to co-localize with progranulin [20,23,24]. Intriguingly, expression of TMEM106B as compared to control results in increased intracellular progranulin [20,23,24], and changes progranulin’s apparent subcellular compartmentalization as visualized by immunofluorescence microscopy [24].

While studies to date have established TMEM106B as an important risk factor for FTLD-TDP and implicated TMEM106B in progranulin pathways, many basic features of this protein -- including its expression patterns in human brain -- are largely unknown. To further characterize this novel disease-related protein, we investigate here the distribution and appearance of TMEM106B in postmortem human brain samples from normal and disease controls, FTLD-TDP individuals with *GRN* mutations (*GRN* (+) FTLD-TDP), and FTLD-TDP individuals without *GRN* mutations (*GRN* (−) FTLD-TDP).

## Results

### TMEM106B expression by cell type in normal human brain tissue

**Figure 1:**
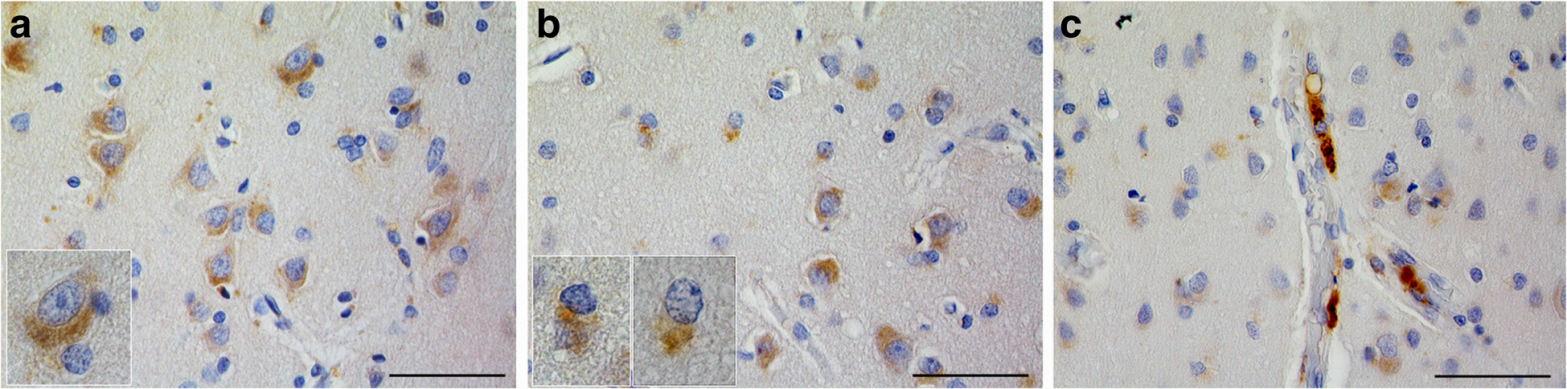
**TMEM106B expression in neurons, glial and endothelial cells or pericytes in cortical specimens from normal controls.** TMEM106B is normally cytoplasmically expressed in neurons **(a)**, glia **(b)**, and endothelial cells or pericytes **(c)**. **(a)** Neuronal staining in cortices from normal human controls demonstrated a perikaryal, polarized cytoplasmic distribution mainly in the cell body and variably extending into processes. **(b)** Glial distribution of TMEM106B similarly demonstrated an asymmetric cytoplasmic distribution. **(c)** A subset of endothelial cells or pericytes demonstrated intense cytoplasmic expression of TMEM106B. Sections were stained with the anti-TMEM106B polyclonal antibody N2077 [24]. Scale bar represents 50 um.

**Figure 2:**
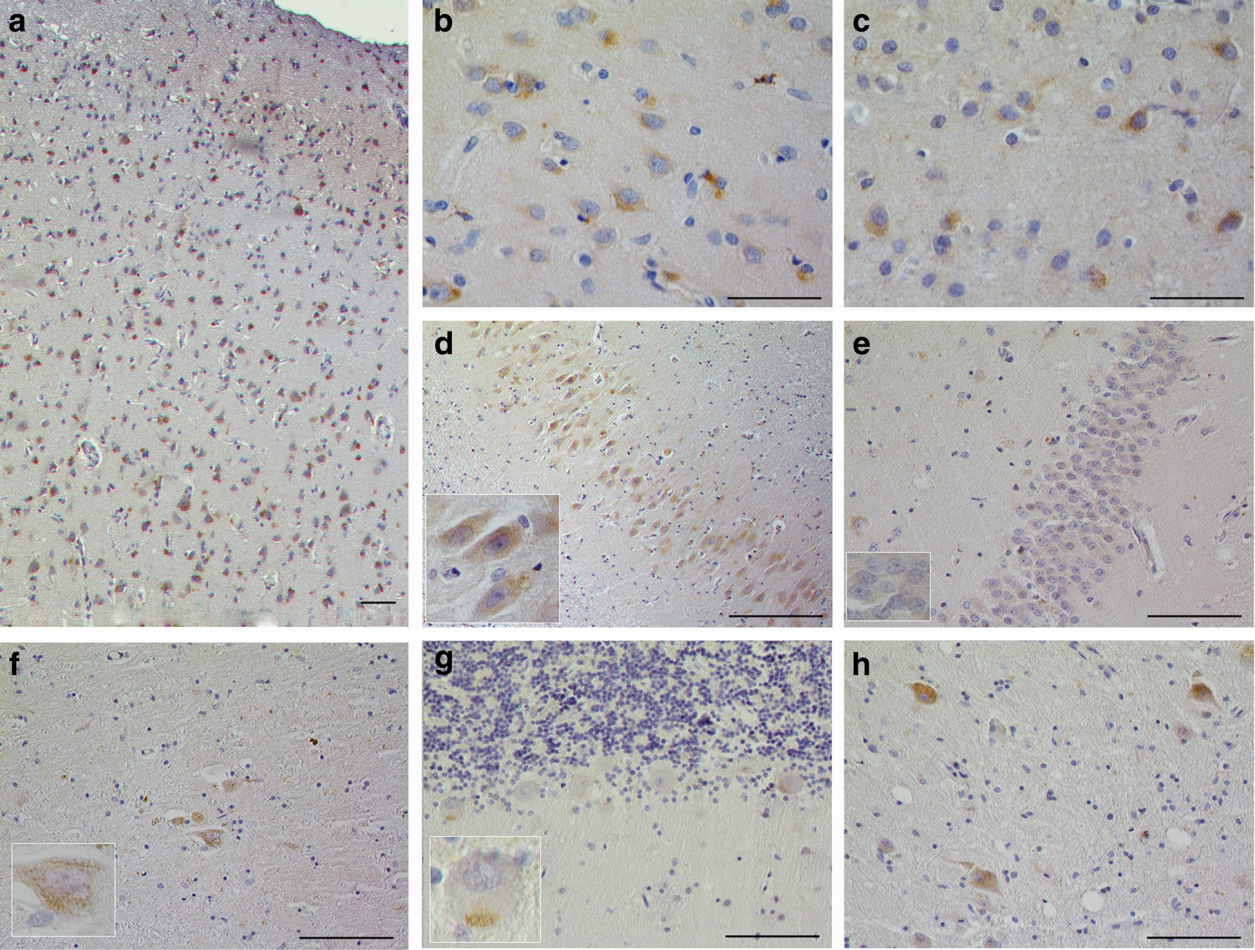
**TMEM106B protein expression in brain tissue from normal controls.** TMEM106B protein expression is found throughout all layers of neocortex. Frontal cortex is shown in **(a)**. TMEM106B neuronal and glial staining pattern is similar in both histopathologically affected areas of the brain (**b**, frontal cortex) and in areas of the brain that are relatively spared of TDP-43 pathology **(c**, occipital cortex**)**. In the hippocampus, the pyramidal neurons of Ammon’s horn show positive TMEM106B immunoreactivity **(d)**, whereas those of the dentate gyrus do not **(e)**. Lentiform nucleus sections demonstrated very rare neuronal staining **(f)**. There was minimal staining of the Purkinje cells of the cerebellum; rarely, cells demonstrated a cytoplasmic, granular staining, as highlighted in **(g)**. Neurons of the deep cerebellar nuclei demonstrated diffuse cytoplasmic TMEM106B **(h)**. Sections were stained with the anti-TMEM106B polyclonal antibody N2077 [24]. Scale bar represents 50 um.

We began by characterizing TMEM106B expression in normal human brain tissue. As shown in Figure 1, we found that TMEM106B is expressed in neurons, glia, and in cells surrounding blood vessels in frontal and occipital cortical samples from normal controls. Specifically, TMEM106B is a cytoplasmic protein that assumes a polarized, perikaryal distribution in neurons (Figure 1a). Glial cells also demonstrate TMEM106B in an asymmetric, polarized pattern within the cytoplasm (Figure 1b). Finally, occasional robust TMEM106B immunoreactivity was observed peri-vascularly, in endothelial cells or pericytes (Figure 1c). TMEM106B immunoreactivity in neurons and glia was observed throughout all layers of neocortex, with prominent expression in the pyramidal neurons of layers 3–5 (Figure 2a).

### TMEM106B expression by brain region in normal human brain tissue

We next evaluated whether TMEM106B expression and appearance varies by brain region. In occipital cortex, a region of the brain relatively spared from TDP-43 pathology, neurons and glia had a similar perikaryal, cytoplasmic pattern of TMEM106B expression when compared to neurons and glia of frontal cortex, a brain region which typically displays a heavy burden of TDP-43 pathology (Figure 2b and c) [30].

In the hippocampus, however, whereas TMEM106B expression was clearly seen in the pyramidal neurons of Ammon’s horn (Figure 2d), no significant staining of the dentate gyrus was observed (Figure 2e), suggesting neuronal subtype specificity of TMEM106B expression. Lentiform nucleus sections from normal controls showed minimal TMEM106B expression (Figure 2f); additionally, neurons of the nucleus basalis of Meynert had little to no staining (not pictured). In cerebellar sections, Purkinje cells demonstrated little TMEM106B expression (Figure 2g), and neurons of the granular layer did not stain for TMEM106B (Figure 2g). In contrast, neurons of the deep cerebellar nuclei showed diffuse TMEM106B immunoreactivity with varying degrees of granularity (Figure 2h).

In summary, we observed variability in TMEM106B expression by neuronal subtype. However, TMEM106B expression did not demonstrate obvious differences in neocortical regions vulnerable to neurodegeneration, compared to those relatively resilient to neurodegeneration, in FTLD-TDP.

### TMEM106B expression in FTLD-TDP brain

Given the putative role of TMEM106B in FTLD-TDP, we stained frontal cortex, occipital cortex, cerebellar, hippocampal, and lentiform nucleus sections from individuals with *GRN* (+) FLTD-TDP, *GRN* (−) FTLD-TDP, and normal controls. In addition, we included FTLD-tau, and Alzheimer’s disease (AD) brain samples as non-FTLD-TDP disease controls.

While FTLD-TDP is characterized by neuronal cytoplasmic inclusions (NCI) and (depending on histological subtype) neuronal intranuclear inclusions (NII) of TDP-43, these TDP-43-containing pathological inclusions did not contain TMEM106B. Furthermore, TMEM106B did not appear to form pathological inclusions of any type in the eleven FTLD-TDP cases investigated here. Comparing normal and disease-affected specimens, however, we noted greater variability in the appearance of TMEM106B cytoplasmic staining among the disease cases. Specifically, in neurons, cytoplasmic TMEM106B ranged from an organized perikaryal distribution to a disordered phenotype in which TMEM106B was expressed diffusely throughout the cell body and even extended into neuronal processes.

**Figure 3:**
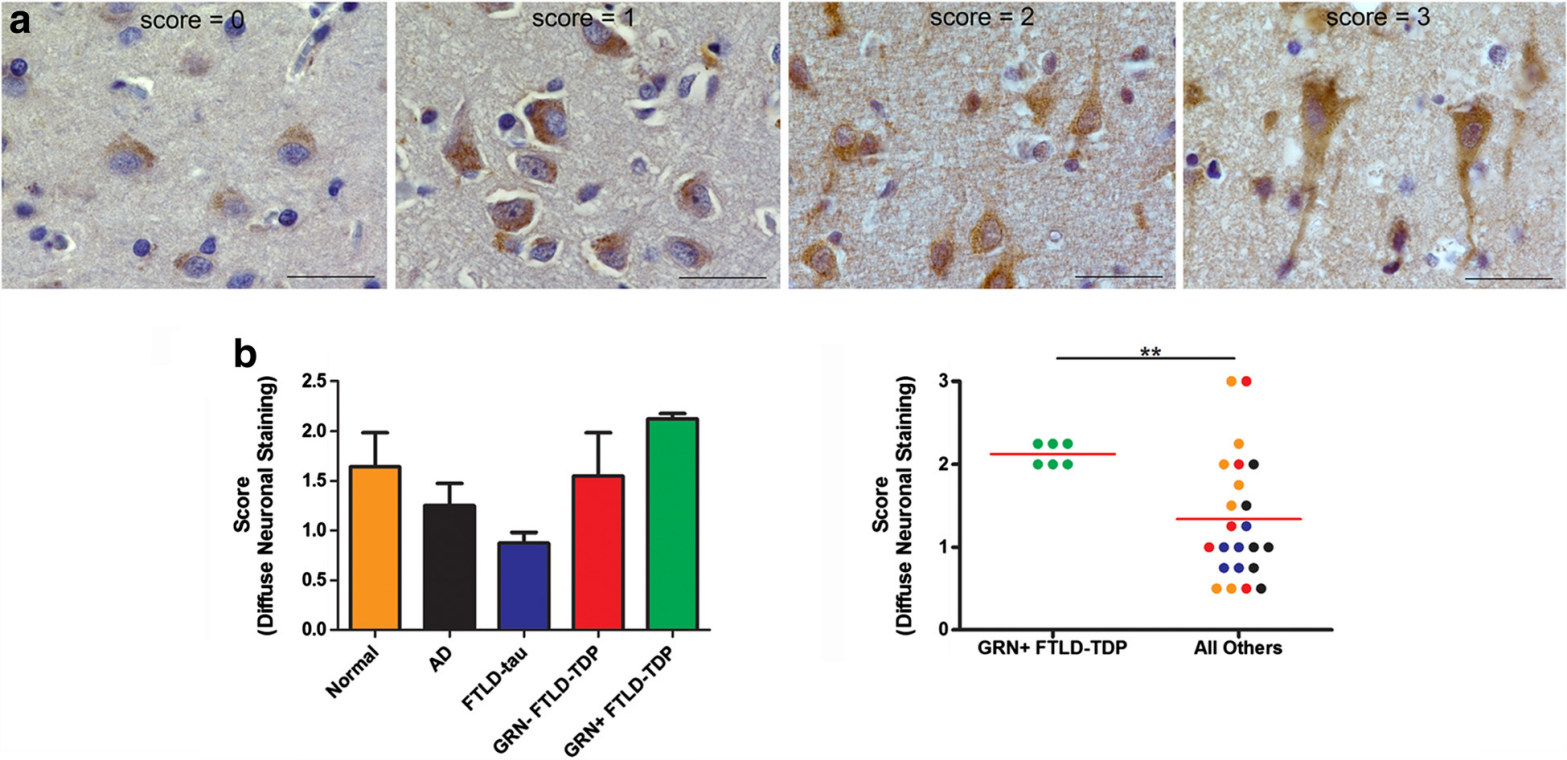
**Scoring of neuronal TMEM106B protein expression. (a)** Scoring schema used to grade severity of disorganization of neuronal TMEM106B expression. Scores of 0 were assigned to sections in which almost all neurons displayed cytoplasmic TMEM106B expression with a vesicular pattern exhibiting a polarized quality. Nuclear boundaries were clear. Scores of 1 were assigned to sections in which a sizeable number of neurons displayed more diffuse TMEM106B staining dispersed more widely in the cytoplasm, but still delimited to the soma. Polarity was still usually maintained. Scores of 2 were assigned to sections in which most neurons recapitulated the characteristics of a score of 1. However, these sections also contained *rare*, non-degenerating neurons which displayed highly disorganized and diffuse TMEM106B staining throughout the cytoplasm with extension into processes. Scores of 3 were assigned to sections in which *numerous* neurons displayed highly disorganized and diffuse TMEM106B staining, with extension into processes. Scale bar represents 30 um. **(b)** Shown is the average scoring of the degree of diffuse neuronal TMEM106B expression by two independent, blinded scorers for N2077-stained human frontal cortical samples. Normal cases n = 7; Alzheimer’s disease n = 5; FTLD-tau n = 6, *GRN* (−) FTLD-TDP n = 5, *GRN* (+) FTLD-TDP n = 6. The colors in the dot plot correspond to the groups delineated in the bar graph. Weighted kappa = 0.44. *GRN* (+) FTLD-TDP cases demonstrated more disorganized patterns of TMEM106B expression (p = 0.005 for Mann–Whitney test).

To further characterize these differences, we semi-quantitatively rated specimens based on their degree of apparent TMEM106B disorganization and loss of subcellular localization using an ordinal scale ranging from 0 (most polarized/organized) to 3 (most diffuse/disorganized). Specifically, two individuals blinded to disease status rated 29 frontal cortex samples for patterns of TMEM106B staining, as described in Figure 3a (normal controls n = 7; AD n = 5; FTLD-tau n = 6, *GRN* (+) FTLD-TDP n = 6, *GRN* (−) FTLD-TDP n = 5).

**Figure 4:**
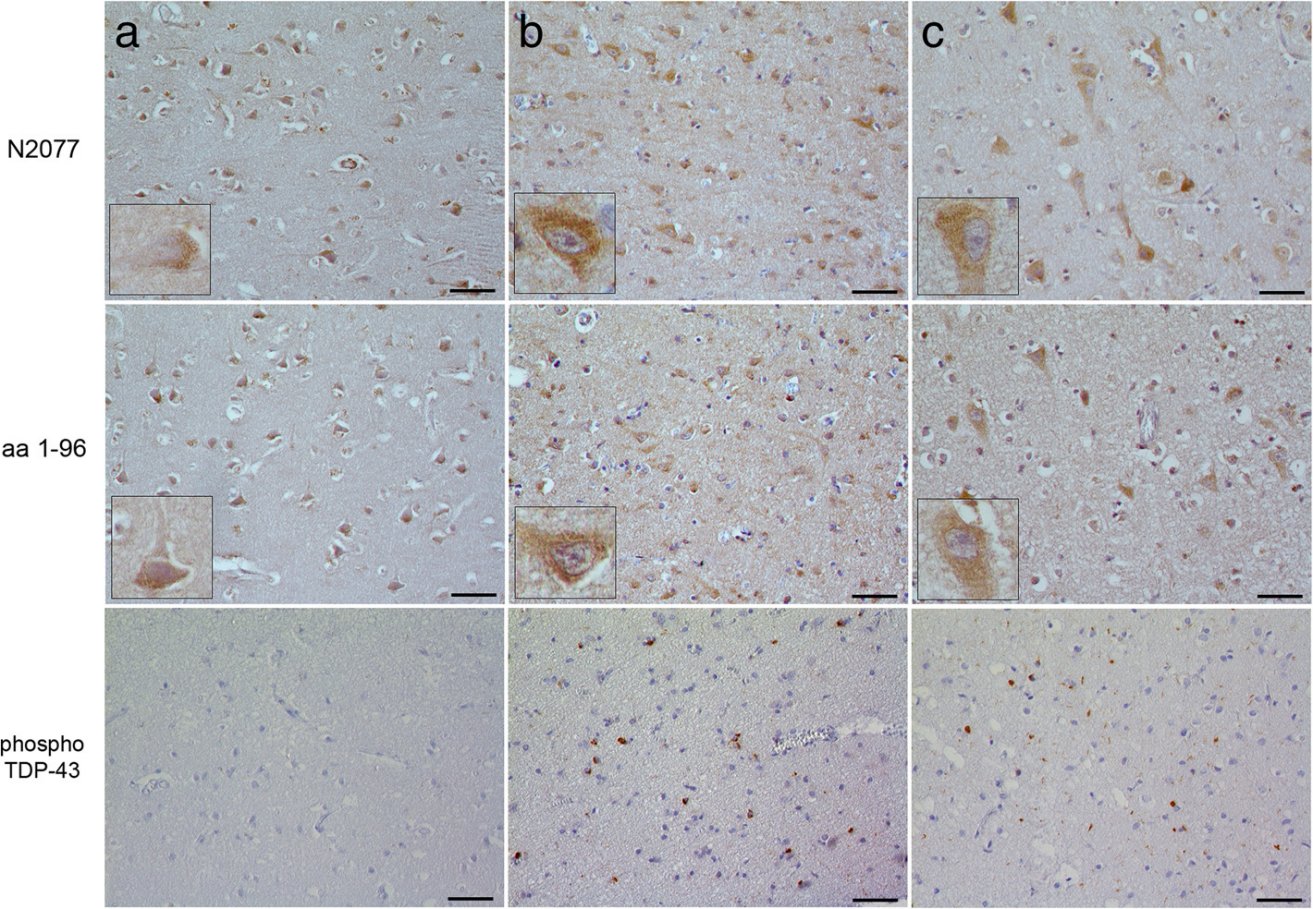
**TMEM106B expression is more disorganized in neurons from *GRN *(+) FTLD-TDP cases, despite comparable levels of TDP-43 pathology.** Representative frontal cortical sections from a normal control **(a)**, *GRN* (−) FTLD-TDP **(b)**, and *GRN* (+) FTLD-TDP **(c)**. Both the N2077 antibody (top row) [24] and a different polyclonal antibody (middle row) raised against the N-terminus (amino acids 1–96) of TMEM106B [23] show similar patterns of immunoreactivity on serial sections from the same cases. *GRN* (+) FTLD-TDP cases showed more disorganized TMEM106B expression than *GRN* (−) FTLD-TDP cases, despite similar degrees of TDP-43 pathology, as indicated by staining against pathological, phosphorylated forms of TDP-43 (bottom row). Scale bar represents 50 um.

Inter-rater reliability was moderately high (weighted kappa = 0.44). Moreover, as shown in Figure 3b, *GRN* (+) FTLD-TDP cases showed the most disorganized patterns of TMEM106B staining, with an average score (2.125) that was significantly greater when compared to all other cases (Mann–Whitney test, p = 0.005). Moreover, while TMEM106B expression rarely extended into neuronal processes for normal controls, FTLD-tau, AD, or *GRN* (−) FTLD-TDP cases, in every *GRN* (+) FTLD-TDP case, we observed TMEM106B expression extending into neuronal processes even in otherwise healthy-appearing neurons. Staining sections with a second TMEM106B antibody demonstrated similar results (Figure 4). Furthermore, TDP-43 pathology did not differ significantly between *GRN* (+) FTLD-TDP and *GRN* (−) FTLD-TDP cases (Figure 4). Of note, *GRN* (−) FTLD-TDP cases used in this study were matched by histopathological subtype to *GRN* (+) FTLD-TDP cases; all were FTLD-TDP Type A cases [31].

Thus, TMEM106B expression differs significantly in frontal cortex neurons of *GRN* (+) FTLD-TDP brain. Specifically, in this genetic subtype, TMEM106B is diffusely expressed throughout the neuronal cytoplasm, with frequent extension into neuronal processes.

## Discussion

In this study, we have provided an initial characterization of TMEM106B protein expression in normal, *GRN* (−) FTLD-TDP, and *GRN* (+) FTLD-TDP human brain, as well as other neurodegenerative disease controls. We find that TMEM106B is normally expressed in the cytoplasm of neurons, glia, and peri-vascular endothelial cells or pericytes, although there may be differences based on neuronal subtype. Unlike many neurodegenerative disease-related proteins, TMEM106B does not form pathological inclusions in diseased brain. Instead, we show that neurons in *GRN* (+) FTLD-TDP cases exhibit more disorganized cytoplasmic TMEM106B expression than normal or disease controls. Specifically, TMEM106B expression in these cases demonstrates loss of polarity as well as subcellular compartmentalization.

In immortalized cell lines and primary cortical neurons, we and others have shown that TMEM106B is localized to endosomes or lysosomes [20,22-24]. We note that the pattern of TMEM106B staining in normal human brain tissue is compatible with this subcellular localization as well, although further studies using double-label immunofluorescence would be needed to definitively demonstrate this.

The present finding that *GRN* (+) FTLD-TDP cases exhibit significantly different patterns of TMEM106B expression is intriguing. It is unlikely that this finding is due to neurodegeneration alone, since disease controls (FTLD-tau, AD) showed the least disorganization. Moreover, *GRN* (−) FTLD-TDP cases with similar patterns of TDP-43 pathology did not demonstrate TMEM106B expression extending into neuronal processes, suggesting that this effect is specific to the *GRN* (+) FTLD-TDP genetic subtype. We have previously shown that *GRN* (+) FTLD-TDP has a distinct global mRNA expression profile [32], suggesting that distinct pathophysiological mechanisms may exist in this molecularly defined subgroup. Moreover, recent evidence implicates TMEM106B in *GRN* (+) FTLD-TDP pathways. Specifically, we have previously shown that TMEM106B may be expressed at higher levels in *GRN* (+) FTLD-TDP brain [24], which is consistent with the histopathological pattern described here of TMEM106B expression throughout the neuronal cytoplasm in these genetic cases. Secondly, *TMEM106B* may act as a genetic modifier among *GRN* mutation carriers, influencing age at disease onset and levels of circulating progranulin [26,27]. Finally, we and others have recently demonstrated that over-expression of TMEM106B affects endo-lysosomal appearance and function as well as the distribution of progranulin in intracellular and extracellular compartments [20,23,24].

In this context, the current study provides further evidence of a relationship between TMEM106B and progranulin, although the directionality of this relationship is unclear. The observation that *GRN* mutation carriers exhibit disordered TMEM106B expression suggests that abnormalities in progranulin can influence TMEM106B expression patterns, whereas the TMEM106B over-expression studies suggest that TMEM106B levels affect progranulin. One possibility to reconcile these findings is that a feedback loop exists between TMEM106B and progranulin in the pathogenesis of FTLD-TDP. Additional studies to investigate this possibility would be a valuable addition to the data presented here.

Our current study has several limitations. First, our sample size of 29 cases may not adequately represent the full range of TMEM106B expression that might exist in a larger sample size. However, even with this small sample size, we were able to detect a significant difference in TMEM106B expression in *GRN* (+) FTLD-TDP. Second, the use of postmortem brain samples limits our ability to interpret the current finding, since non-specific effects due to postmortem interval, disease duration, cell loss and gliosis could confound our results. Finally, samples used here were not strictly age- and gender-matched among groups. However, the *GRN* (+) FTLD-TDP group did not differ significantly from the other groups in these respects (*t*-test p = 0.612 for age comparison, chi-square p = 0.775 for sex comparison), decreasing the possibility that these demographic variables may account for the observed effect.

## Conclusions

In conclusion, we have provided the first histological characterization of TMEM106B expression in multiple regions of pathological and normal human brain. Our data add to the growing body of evidence that TMEM106B and progranulin may be linked mechanistically in the pathogenesis of FTLD-TDP. Further characterization of this new FTLD-TDP risk factor, as well as its interactions with progranulin, may open up new avenues for the development of disease-modifying therapies.

## Methods

### Brain samples

Human postmortem brain samples were obtained from the University of Pennsylvania Center for Neurodegenerative Disease Brain Bank under IRB approval. These comprised samples from normal individuals (n = 7), as well as individuals with FTLD-TDP (n = 11), FTLD-tau (n = 6), and Alzheimer’s disease (AD, n = 5). Regions sampled included midfrontal cortex, occipital cortex, cerebellum, lentiform nucleus, and hippocampus. See Additional file 1: Table S1 for a detailed list of cases. Histopathological subtyping for FTLD-TDP was performed according to established criteria [31]. Genetic testing for *C9orf72* expansions, *GRN* mutations, and *MAPT* mutations was performed as previously described [33,34]. One FTLD-TDP case was found to harbor a *C9orf72* expansion -- TMEM106B expression in this case did not appear atypical for the *GRN* (−) FTLD-TDP group. In addition, 6 FTLD-TDP cases had *GRN* mutations, and no cases had *MAPT* mutations.

### Immunohistochemistry

Formalin-fixed, paraffin-embedded 6 μm sections from various brain regions were cleared in a descending ethanol series then blocked with 3% H2O2/MeOH for 30 minutes. After washing, sections were immersed in Antigen Unmasking Solution (Vectashield) and microwaved 1 × 10 minutes at 50% power, then 2 × 6 minutes at 50% power. Slides were allowed to cool to room temperature, then washed with 0.1 M Tris buffer, pH 7.6 (Tris) for five minutes. Sections were blocked in Tris + 2% FBS, pH 7.6 (Tris/FBS) for five minutes, before overnight incubation at 4°C with primary antibody (see Additional file 1: Table S2 for antibody conditions). Specimens were immersed in Tris buffer x 5 minutes, followed by Tris/FBS x 5 minutes. Biotinylated goat anti-rabbit secondary antibody (Vectashield) was applied, and samples were incubated at room temperature in a humidified chamber for one hour. Samples were washed briefly in Tris. VECTASTAIN AB solution (Vector Labs) made up in Tris/FBS was applied to the samples and incubated for one hour at room temperature. Slides were then incubated with ImmPACT DAB solution (Vector Labs) for 2–8 minutes until desired stain intensity was achieved. Specimens were rinsed briefly with Tris, followed by dH2O and then counterstained with Harris’ hematoxylin (Thermo-Shandon) for 10–30 seconds. Slides were washed in running tap water for 5 minutes. Coverslips were sealed with Cytoseal (Thermo Scientific) and slides were allowed to dry for at least one hour.

TMEM106B antibodies used in this manuscript were N2077, a previously validated [24] polyclonal rabbit antibody directed at amino acids 4–19 of TMEM106B (a peptide sequence specific to TMEM106B). Additional data supporting the specificity of N2077 for TMEM106B are provided in Additional file 1: Figure S1. A second polyclonal rabbit antibody raised against amino acids 1–96 of TMEM106B was used to verify results; this second antibody has been previously validated as well [23].

### Semi-quantitative assessment of TMEM106B expression

Two independent, blinded observers (MML and ACP) scored stained specimens from normal controls (n = 7), as well as from patients with Alzheimer’s disease (n = 5), FTLD-tau (n = 6), *GRN* (+) FTLD-TDP (n = 6), and *GRN* (−) FTLD-TDP (n = 5), assessing for the nature and degree of neuronal staining. Specimens were assigned scores of 0–3 based on an ordinal scale representing increasing loss of subcellular localization and polarity. Representative images and scoring criteria are described in the Results section.

## Competing interests

The authors declare that they have no competing interests.

## Authors’ contributions

JB performed experiments, intepreted the data, and drafted the manuscript. EA performed experiments and revised the manuscript for experimental content. ML analyzed data and revised the manuscript for neuropathological content. MG recruited study participants and revised the manuscript for clinical content. VVD performed genetic screening and revised the manuscript for genetic content. FH developed reagents and revised the manuscript for experimental content. VL and JQT banked and provided neuropathological specimens and edited the manuscript for neuropathological content. ACP conceived of and supervised the study, analyzed data, and drafted the manuscript. All authors read and approved the final manuscript.

## Supplementary Material

Additional file 1: Table S1 Characteristics of cases and brain regions evaluated for TMEM106B expression. **Table S2**. Antibodies and conditions used for immunohistochemical staining. **Figure S1**. Validation of TMEM106B antibody. Click here for file
