# Supplementary material for: Expression of TMEM106B, the frontotemporal lobar degeneration-associated protein, in normal and diseased human brain
Source: Acta Neuropathol Commun. 2013 Jul 11;1:36. doi: 10.1186/2051-5960-1-36 (PMC3893524; doi:10.1186/2051-5960-1-36)
Supplement: Additional file 1: Table S1 — Characteristics of cases and brain regions evaluated for TMEM106B expression. Table S2. Antibodies and conditions used for immunohistochemical staining. Figure S1. Validation of TMEM106B antibody. [file 2051-5960-1-36-S1.docx]

**SUPPLEMENTARY MATERIALS**

**Manuscript: Expression of TMEM106B, the frontotemporal lobar degeneration-associated protein, in normal and diseased human brain**

**INCLUDES:**

**2 Additional file Tables**

**1 Additional Figure**

| Case | Brain Regions Evaluated | Diagnosis | GRN mutation | Other mutation | Age | Gender |
| --- | --- | --- | --- | --- | --- | --- |
| 1 | frontal, hippocampal | AD |  |  | 65 | f |
| 2 | frontal | AD |  |  | 87 | m |
| 3 | frontal | AD |  |  | 87 | m |
| 4 | frontal | AD |  |  | 70 | f |
| 5 | frontal | AD |  |  | 63 | f |
| 6 | frontal | FTLD-tau |  |  | 66 | m |
| 7 | frontal | FTLD-tau |  |  | 66 | m |
| 8 | frontal | FTLD-tau |  |  | 82 | f |
| 9 | frontal | FTLD-tau |  |  | 78 | m |
| 10 | frontal | FTLD-tau |  |  | 87 | m |
| 11 | frontal | FTLD-tau |  |  | 76 | m |
| 12 | frontal, occipital, cerebellar, hippocampal, lentiform | FTLD-TDP GRN (-) |  |  | 49 | f |
| 13 | frontal | FTLD-TDP GRN (-) |  |  | 48 | m |
| 14 | frontal | FTLD-TDP GRN (-) |  |  | 53 | m |
| 15 | frontal | FTLD-TDP GRN (-) |  |  | 77 | f |
| 16 | frontal | FTLD-TDP GRN (-) |  | *C9ORF72* | 73 | f |
| 17 | frontal, occipital, cerebellar, hippocampal, lentiform | FTLD-TDP GRN (+) | c.1252C>T |  | 62 | f |
| 18 | frontal | FTLD-TDP GRN (+) | c.1477C>T |  | 65 | m |
| 19 | frontal | FTLD-TDP GRN (+) | c.348A>C |  | 78 | m |
| 20 | frontal | FTLD-TDP GRN (+) | c.1179+2T>C |  | 61 | m |
| 21 | frontal | FTLD-TDP GRN (+) | c.911G>A |  | 78 | f |
| 22 | frontal | FTLD-TDP GRN (+) | c.1252C>T |  | 68 | f |
| 23 | frontal | Normal |  |  | 56 | f |
| 24 | frontal | Normal |  |  | 83 | m |
| 25 | frontal | Normal |  |  | 82 | f |
| 26 | frontal | Normal |  |  | >90 | f |
| 27 | frontal | Normal |  |  | 55 | f |
| 28 | frontal, occipital, cerebellar, hippocampal, lentiform | Normal |  |  | 68 | f |
| 29 | frontal, occipital, cerebellar, hippocampal, lentiform | Normal |  |  | 85 | f |

**Additional file 1: Table S1. Characteristics of cases and brain regions evaluated for TMEM106B expression.** GRN mutations were present in cases 17-22. Nomenclature follows

cDNA sequence NM_002087.2; all mutations are believed to be pathogenic (<http://www.molgen.ua.ac.be/admutations/>). Case 16 carries a *C9ORF72* expansion -- this case was rated a "2" by both scorers for TMEM106B staining pattern. No cases had *MAPT* mutations. All FTLD-TDP cases were FTLD-TDP Type A cases.

| Antibody | Antigen | Final concentration |
| --- | --- | --- |
| N2077 | TMEM106B (aa 4-19) | 1 ug/ml |
| aa 1-96 TMEM106B | TMEM106B (aa 1-96) | 1:750 |
| p409/410 | TDP-43 (phosphorylated at aa 409/410) | 1:500 |

**Additional file 1: Table S2. Antibodies and conditions used for immunohistochemical staining.**

**Additional file 1: Figure S1: Validation of TMEM106B antibody**

**
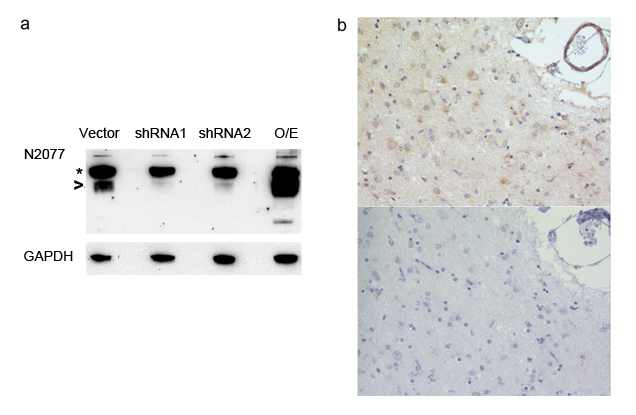
**

(a) The 75kDa band (arrowhead) corresponding to endogenous TMEM106B decreases when cells are transfected with two different shRNAs targeting TMEM106B. Transfection of our tagged overexpression construct (O/E), TMEM-FLAG, results in bands at 75 and 40 kDa, as previously described (*denotes a non-specific band) [24]. (b) Preincubation of specimens with the immunizing peptide (aa 4-19 TMEM106B) results in loss of TMEM106B immunoreactivity in serial sections from frontal lobe.
